# Structural Insights Into Tautomeric Dynamics in Nucleic Acids and in Antiviral Nucleoside Analogs

**DOI:** 10.3389/fmolb.2021.823253

**Published:** 2022-01-25

**Authors:** Bogdan I. Fedeles, Deyu Li, Vipender Singh

**Affiliations:** ^1^ Departments of Chemistry and Biological Engineering and Center for Environmental Health Sciences, Massachusetts Institute of Technology, Cambridge, MA, United States; ^2^ Department of Pharmaceutical Sciences, College of Pharmacy, University of Rhode Island, Kingston, RI, United States; ^3^ Department of Biochemistry and Biophysics, Novartis Institute of Biomedical Research, Cambridge, MA, United States

**Keywords:** Tautomerism, ribozymes, riboswitches, mutagenesis, COVID-19, antivirals, nucleoside analogs therapy, spontaenous mutations

## Abstract

DNA (2′-deoxyribonucleic acid) and RNA (ribonucleic acid) play diverse functional roles in biology and disease. Despite being comprised primarily of only four cognate nucleobases, nucleic acids can adopt complex three-dimensional structures, and RNA in particular, can catalyze biochemical reactions to regulate a wide variety of biological processes. Such chemical versatility is due in part to the phenomenon of nucleobase tautomerism, whereby the bases can adopt multiple, yet distinct isomeric forms, known as tautomers. For nucleobases, tautomers refer to structural isomers that differ from one another by the position of protons. By altering the position of protons on nucleobases, many of which play critical roles for hydrogen bonding and base pairing interactions, tautomerism has profound effects on the biochemical processes involving nucleic acids. For example, the transient formation of minor tautomers during replication could generate spontaneous mutations. These mutations could arise from the stabilization of mismatches, in the active site of polymerases, in conformations involving minor tautomers that are indistinguishable from canonical base pairs. In this review, we discuss the evidence for tautomerism in DNA, and its consequences to the fidelity of DNA replication. Also reviewed are RNA systems, such as the riboswitches and self-cleaving ribozymes, in which tautomerism plays a functional role in ligand recognition and catalysis, respectively. We also discuss tautomeric nucleoside analogs that are efficacious as antiviral drug candidates such as molnupiravir for coronaviruses and KP1212 for HIV. The antiviral efficacy of these analogs is due, in part, to their ability to exist in multiple tautomeric forms and induce mutations in the replicating viral genomes. From a technical standpoint, minor tautomers of nucleobases are challenging to identify directly because they are rare and interconvert on a fast, millisecond to nanosecond, time scale. Nevertheless, many approaches including biochemical, structural, computational and spectroscopic methods have been developed to study tautomeric dynamics in RNA and DNA systems, and in antiviral nucleoside analogs. An overview of these methods and their applications is included here.

## Introduction

**FIGURE 1:**
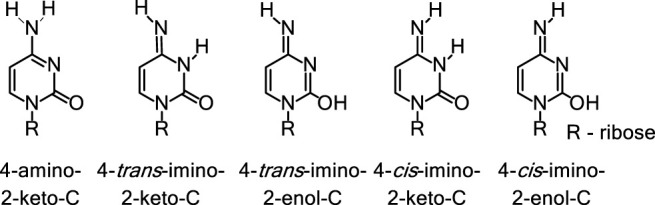
Prototropic tautomerism in cytosine. Nucleic acid bases contain keto, amino, or both functional groups. These groups often participate in keto-enol and amino-imino tautomerism as shown here for the native base cytosine.

**FIGURE 2:**
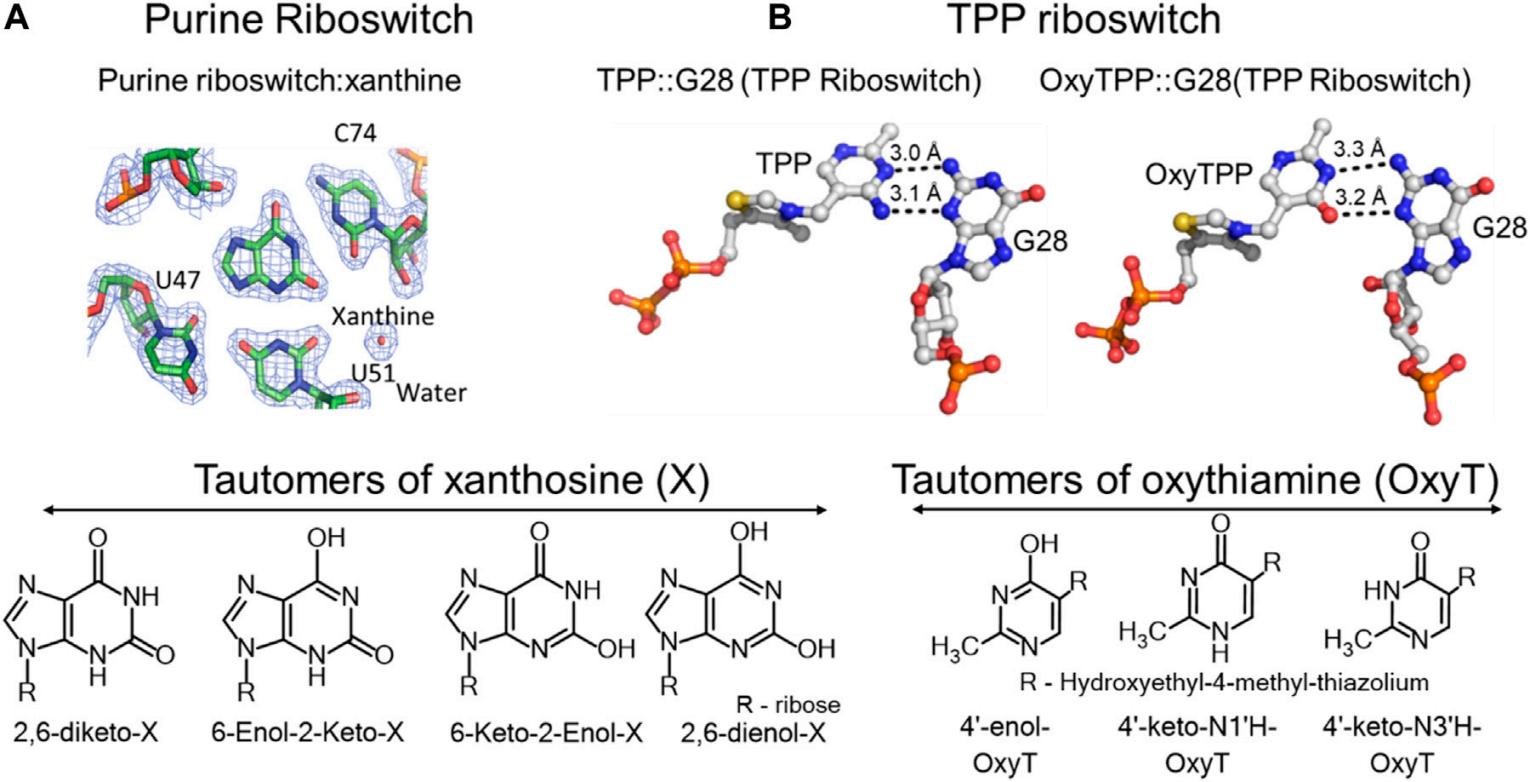
Tautomerism in riboswitches. **(A)** Top left, crystal structure of the purine riboswitch with xanthine in its binding pocket interacting with carbonyl oxygens of C74, U47 and U51, and a water molecule. Bottom left, all possible tautomeric forms of xanthosine. **(B)** Crystal structures of the TPP riboswitch showing interactions of its binding site G28 (guanosine at the 28th position) with the thiamine pyrophosphate (TPP) and oxythiamine pyrophosphate (OxyTPP) ligands. Bottom, tautomeric forms of oxythiamine identified by NMR and vibrational spectroscopy (Singh et al., 2014). The figure is adapted from reference (Singh et al., 2014) (CC BY 4.0).

**FIGURE 3:**
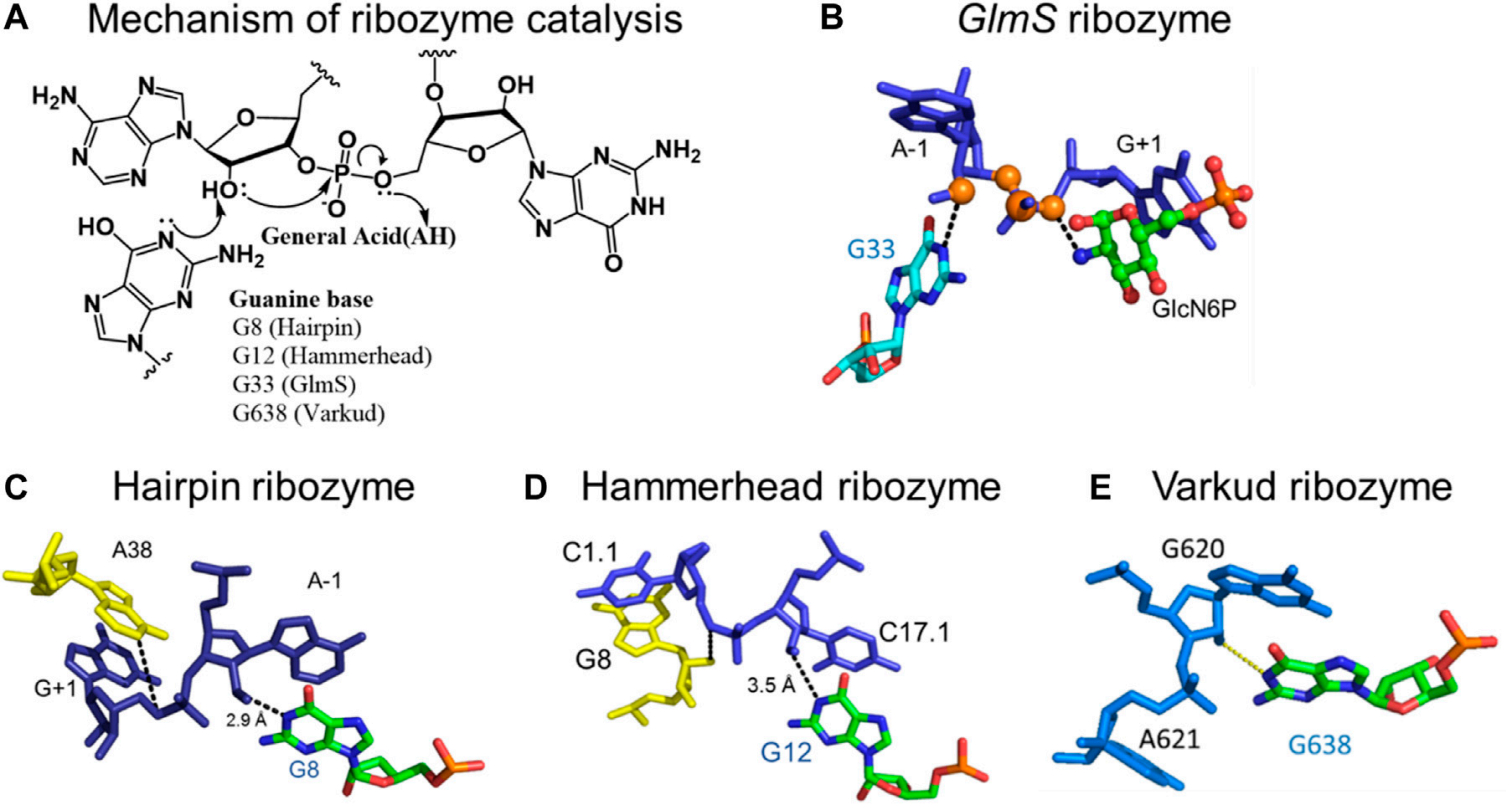
Mechanism of the nucleolytic reaction catalyzed by small self-cleaving ribozymes. **(A)** Acid-base catalytic mechanism of small self-cleaving ribozymes and the positions of participating catalytic guanosines in the active sites of the respective ribozymes. **(B)** Catalytic guanosine (G33) in the active site of the *glmS* ribozyme, in close proximity to the 2′-hydroxyl of the A-1 nucleotide adjacent to the scissile phosphodiester bond. **(C)** The N1 of catalytic guanosine (G8) in the hairpin ribozyme in close proximity to the 2′-hydroxyl of the A-1 nucleotide. **(D)** The N1 of catalytic guanosine (G12) in the hammerhead ribozyme in close proximity to the 2′-hydroxyl of the A-1 nucleotide. **(E)** The N1 of the catalytic guanosine (G698) in the Varkud Satellite ribozyme in close proximity the 2′-hydroxyl of A-1 nucleotide. Figure 3E was shared by Joe Piccirilli’s laboratory at the University of Chicago (Suslov et al., 2015), and parts of the figure are adapted from reference (Singh et al., 2014) (CC BY 4.0).

**FIGURE 4:**
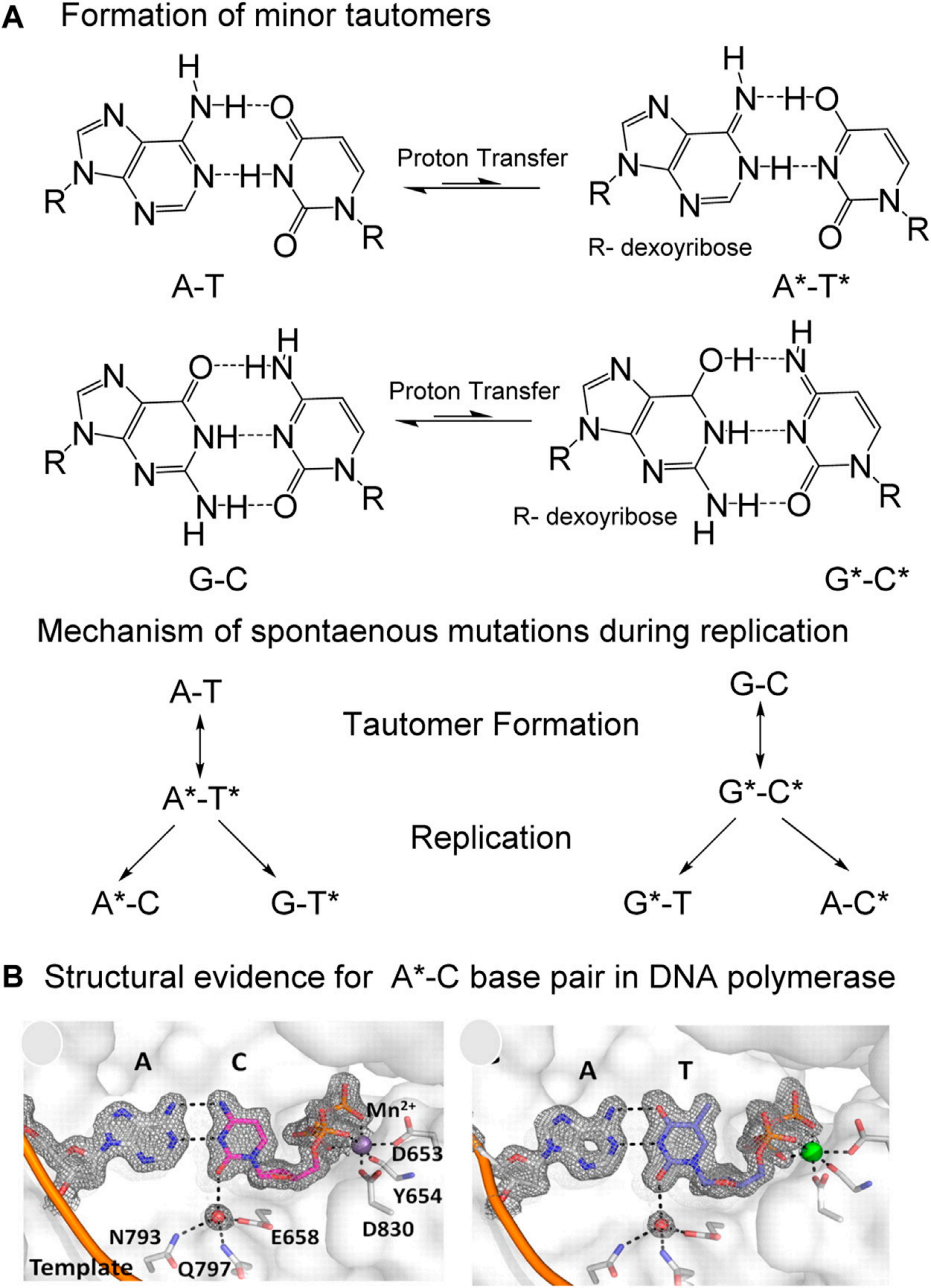
Proposed mechanism by which “spontaneous” mutations are introduced by the inter-strand movement of protons in Watson-Crick (W–C) base pairs during replication. **(A)** Spontaneous transfer of two protons from one strand to another in A-T and G-C base pairs generate minor tautomers, which can form mismatches during replication to cause mutations. **(B)** Structural evidence for the stabilization of A-C base pair in W-C conformation, almost indistinguishable from the A-T base pair in active site of a high fidelity DNA polymerase. Figure is adapted from reference (Wang et al., 2011; Slocombe et al., 2021).

Nucleic acid bases exhibit keto-enol and amino-imino prototropic tautomerism due to the presence of multiple solvent-exchangeable protons (Figure 1) (Watson and Crick, 1953; Topal and Fresco, 1976; Brown et al., 1989; Colominas et al., 1996; Mons et al., 2002). The formation of minor tautomers can increase the overall structural and chemical diversity of nucleic acids, which enables their diverse functions in biology (Topal and Fresco, 1976; Cochrane and Strobel, 2008a; Singh et al., 2015). For example, many self-cleaving ribozymes (RNA enzymes) and some riboswitches (RNA aptamers) are proposed to utilize tautomerism to perform their biological function (Figure 2, Figure 3) (Singh et al., 2015; Singh et al., 2014). Formation of minor tautomers in DNA, at low frequency, is proposed to contribute to the phenomenon of ‘spontaneous mutagenesis’, which denotes the background level of mutations that appear during the replication of undamaged DNA (Watson and Crick, 1953; Topal and Fresco, 1976; Wang et al., 2011; Rangadurai et al., 2020). These mutations are thought to arise due, in part, to the altered base pairing properties of minor tautomers (Figure 4) (Watson and Crick, 1953; Topal and Fresco, 1976; Wang et al., 2011).

**FIGURE 5:**
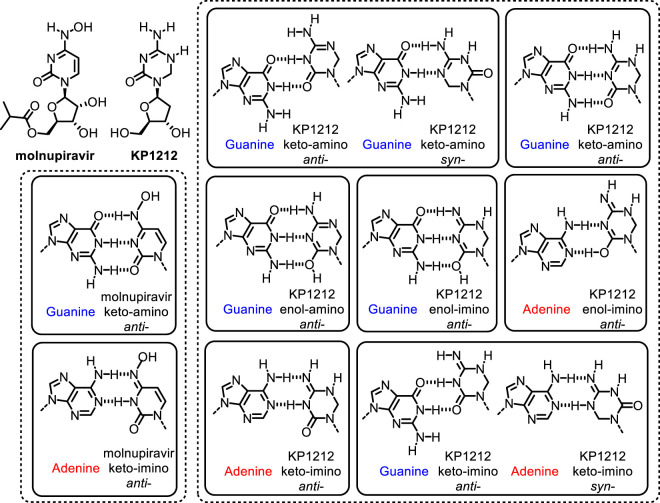
Proposed tautomeric structures of antiviral drugs molnupiravir and KP1212, and base pairing properties during replication in SARS-CoV-2 and HIV viruses, respectively (Li et al., 2014; Kabinger et al., 2021).

Therapeutics based on tautomeric nucleoside analogs have proven effective as antiviral agents against a range of retro- and ribo-viruses, including influenza (Delang et al., 2018), hepatitis-C-virus (HCV) (Crotty et al., 2001), human immunodeficiency virus (HIV) (Li et al., 2014) and coronaviruses, including COVID-19 (Figure 5) (Shannon et al., 2020; Kabinger et al., 2021). The antiviral efficacy of these analogs stems from their ability to exist in multiple tautomeric or rotameric states, which help mutagenize the viral genomes to error catastrophe and even to viral population extinction. The formation of minor tautomers in nucleic acids is a rare event, and their dynamics of interconversion is fast, on a millisecond to nanosecond time scale (Peng et al., 2013; Rangadurai et al., 2019). Recent developments in methods have allowed direct identification and quantification of minor tautomers in nucleic acid bases and in nucleoside/nucleotide analogs (Peng et al., 2011; Li et al., 2014; Singh et al., 2014; Peng et al., 2015; Rangadurai et al., 2019).

Small self-cleaving ribozymes and many riboswitches are proposed to utilize minor tautomeric forms of catalytic residues and ligands to perform their biological functions (Figure 2, Figure 3) (Cochrane et al., 2007; Cochrane and Strobel, 2008a; Cochrane and Strobel, 2008b; Thore et al., 2008; Gilbert et al., 2009; Wilcox and Bevilacqua, 2013; Singh et al., 2014; Singh et al., 2015). Among riboswitches, crystallographic evidence shows that the purine and the thiamine pyrophosphate riboswitches recognize the minor tautomeric forms of their non-natural ligands xanthine and oxythiamine pyrophosphate (OxyTPP), respectively (Figure 2) (Thore et al., 2008; Gilbert et al., 2009; Singh et al., 2015; Singh et al., 2014). Self-cleaving ribozymes such as hammerhead, hairpin, glmS, varkud satellite (VS), hepatitis delta virus (HDV), and twister catalyze nucleolytic intramolecular self-scission reactions (Figure 3) (Rupert and Ferré-D'Amaré, 2001; Martick and Scott, 2006; Cochrane et al., 2007; Cochrane and Strobel, 2008a; Suslov et al., 2015). In these ribozymes, it is proposed that the tautomeric forms of their catalytic guanosines act as a general base to activate the 2′-hydroxyl nucleophile to initiate the cleavage reactions (Klein et al., 2007; Cochrane and Strobel, 2008a).

During DNA replication, formation of minor tautomeric forms of nucleobases could lead to mispairing that generates mutations in the absence of any DNA lesion or other form of DNA damage (Watson and Crick, 1953; Löwdin, 1963; Topal and Fresco, 1976; Wang et al., 2011; Bebenek et al., 2011; Rangadurai et al., 2019; Rangadurai et al., 2020). This phenomenon is thought to explain the appearance of spontaneous mutations during the replication of undamaged DNA. The transient generation of minor tautomers in DNA allows stabilization of mismatches, in the polymerase active site, in conformations that are indistinguishable from the structures of canonical base pairs (Figure 4) (Watson and Crick, 1953; Topal and Fresco, 1976; Wang et al., 2011; Rangadurai et al., 2020).

While assembling their double-helix model of DNA, James Watson and Francis Crick stumbled over the phenomenon of base tautomerism; in fact, their model required that the bases adopt specific tautomeric forms in order to base-pair, with the suggestion that alternate tautomers would lead to mispairing and spontaneous mutations. (Watson and Crick, 1953). These ideas were later formalized by Michael Topal and Jacques Fresco, who described in 1976 the specific pairings between the minor tautomeric forms of canonical nucleic acid bases during replication that are likely to lead to mutations (Topal and Fresco, 1976). Since then, structural studies have shown that the formation of minor tautomers in DNA could allow wobble mismatches, such as the A-C and G-T base pairs, to adopt a geometry similar to that of canonical base pairs in Watson and Crick (W-C) conformations (Figure 4) (Wang et al., 2011; Szymanski et al., 2017). These studies provided structural evidence in support of the rare tautomer hypothesis for spontaneous mutations (Wang et al., 2011). Furthermore, tautomeric dynamics occur on a time scale that is similar to that of DNA replication, suggesting that the rate of rare tautomer formation could determine the probability of base misincorporation during replication (Rangadurai et al., 2019; Rangadurai et al., 2020).

In this review, we will discuss mechanisms involving tautomerism in RNA biology and in the generation of spontaneous mutations during DNA replication. We will also discuss the role of tautomerism in the development of nucleoside/nucleotide analogs based antiviral therapeutics. Specifically, we will focus on tautomerism in the catalysis of small self-cleaving ribozymes and in ligands recognition by riboswitches. We will discuss evidence of tautomerism in DNA and the associated mechanisms that contribute to the generation of spontaneous mutations. We will also review the role of tautomerism in the mechanism by which antiviral drug candidates based on nucleoside analogs, such as molnupiravir (isopropyl ester prodrug of the ribonucleoside analog β-D-N^4^-hydroxycytidine) and KP1212 (5-aza-5,6-dihydro-2′-deoxycytidine), induce lethal mutagenesis in the genomes of SARS-CoV-2 and HIV, respectively. Minor tautomers are rare and their dynamics of interconversion is fast, which makes them challenging to identify in complex macromolecular systems, such as DNA and RNA. In the last section, we will summarize the current, state-of-the-art methods for studying tautomerism in nucleic acids and in antiviral nucleoside analogs, and discuss the future directions of the field.

### Tautomerism in Nucleic Acids

Nucleic acids display keto-enol and amino-imino types of tautomerism (Singh et al., 2015), as shown in Figure 1 using cytosine as an example. This type of tautomerism, called prototropic tautomerism (Laar, 1886; Antonov, 2013) involves the movement of protons in the form of deprotonation at one site and protonation at another site, which is accompanied by a rearrangement of the double bonds in between the two sites. The base pairing complementarity in nucleic acids depends on the position of protons on the Watson-Crick (W-C) interface (Watson and Crick, 1953; Wang et al., 2011). As the positions of protons change between different tautomers (Figure 1) (Singh et al., 2015), the distribution of hydrogen donor and acceptor sites is altered on the W-C face of each base. Therefore, certain minor tautomeric forms of canonical nucleic acid bases can engage in alternative base pairings (Watson and Crick, 1953; Topal and Fresco, 1976; Wang et al., 2011; Singh et al., 2015; Rangadurai et al., 2019; Rangadurai et al., 2020). Since base pairing interactions are predominantly responsible for the biological functions performed by nucleic acids, prototropic tautomerism is expected to influence the biology of both DNA and RNA (Singh et al., 2015). Below we discuss a few notable examples where nucleobase tautomerism is a key contributor or otherwise modulates the biological functions performed by nucleic acids.

#### Tautomerism in RNA Riboswitches

The ligand recognition by the purine and thiamine pyrophosphate riboswitches involves binding to minor tautomeric forms of their non-natural ligands (Figure 2) (Thore et al., 2008; Gilbert et al., 2009; Singh et al., 2014). The purine riboswitch regulates expression of genes involved in the metabolism of adenine and guanine. The regulation is mediated by the conformational change in response to binding to purine ligands (Gilbert et al., 2009; Gilbert et al., 2006). In addition to recognizing adenine and guanine, the purine riboswitch can also bind to metabolites of purines such as xanthine, hypoxanthine and 2,6-diaminopurine (Gilbert et al., 2009; Gilbert et al., 2006). Crystal structures of these ligands bound to the purine riboswitch show that the cytosine at the 74 position (C74) is critical for determining substrate specificity (Figure 2A) (Gilbert et al., 2009). The carbonyl oxygen (O2) of C74 and U51 form hydrogen bonds with the 2-amino functional group of the native ligand guanine (Gilbert et al., 2009), which explain the riboswitch preference for ligands that have a hydrogen bond donor at the 2-position of purines such as guanine and 2,6-diaminopurine (Gilbert et al., 2009; Gilbert et al., 2006). However, the riboswitch can also bind to xanthine, which has a carbonyl group at the 2-position, and hypoxanthine, which lacks any functional group at that positon, albeit with a weaker affinity (Figure 2A) (Gilbert et al., 2009). Xanthine binding has a dissociation constant (K_D_) of 32 μM, three orders of magnitude higher than the nanomolar K_D_s for guanine and 2,6-diaminopurine (Gilbert et al., 2009). Hypoxanthine has 200-fold less affinity compared to guanine (Gilbert et al., 2009). However, the crystal structures of the riboswitch bound to xanthine or guanine ligands reveal no significant structural differences (Gilbert et al., 2009). To rationalize their identical structures, it was proposed that the 2-enol form of xanthine would alleviate the repulsive interactions that exist due to the presence of three negatively charged oxygens in close proximity (Figure 2A) (Singh et al., 2015). This proposal was also consistent with the biochemical observation that xanthine binds the riboswitch at a pH of 6 (K_D_ = 33 µM), but no binding is detected at a higher pH of 8.5 (Gilbert et al., 2009). The enol tautomers are expected to be more stable at lower pH (Gilbert et al., 2009). Despite the observed crystallographic and biochemical evidence in support of the minor 2-enol tautomer, the direct existence of 2-enol xanthine has not been established. This is due, in part, to the lack of sensitive methods for distinguishing keto and enol forms of xanthine in the background overlapping signals from polymeric RNA (Gilbert et al., 2009; Singh et al., 2014).

Tautomerism is also suggested to influence oxythiamine pyrophosphate (OxyTPP) binding to the thiamine pyrophosphate (TPP) riboswitch (Thore et al., 2006; Thore et al., 2008). The TPP riboswitch binds to its natural ligand, TPP, to negatively regulate the expression of genes involved in the biosynthesis and transport of thiamine (Thore et al., 2006). The X-ray structure of the riboswitch with the TPP ligand shows that the amino group at the 4′-position of TPP acts as a hydrogen bond donor to the N3 position of G28 (Figure 2B) (Thore et al., 2006; Thore et al., 2008). The OxyTPP has a carbonyl group at the 4′-position. Yet, under crystalline conditions, its hydrogen bonding interactions to G28 are almost identical to those of TPP (Figure 2B) (Thore et al., 2008). The 4′- position of OxyTPP can only act as a hydrogen bond donor in its enol form, leading to the hypothesis that OxyTPP binds to the riboswitch as an enol tautomer (Thore et al., 2008). The Oxythiamine (Oxy) portion of OxyTPP exists in three tautomeric forms including the proposed 4′-enol tautomer (Singh et al., 2014). Biochemical binding isotope effect (BIE) experiments combined with density functional theory (DFT) calculations performed using O-18 labelled OxyTPP could not unambiguously identify the tautomer of OxyTPP in the binding pocket of the TPP riboswitch (Singh et al., 2014). More sensitive methods, such as NMR relaxation dispersion, low temperature NMR, FTIR (Fourier Transform Infrared Spectroscopy) and 2D-IR (2-Dimentional Infrared Spectroscopy) (discussed in the Methods overview) are needed to unambiguously characterize the tautomeric form of OxyTPP or its binding partner G28 on the TPP riboswitch.

#### Tautomerism in Self-Cleaving Ribozymes

Small self-cleaving ribozymes are proposed to utilize the minor tautomeric form(s) of catalytic guanosines to execute the first step of the self-cleavage reactions (Figure 3) (Cochrane and Strobel, 2008a). As mentioned above, the hammerhead, hairpin, glmS, VS, HDV, and the twister ribozymes are examples of small self-cleaving ribozymes that catalyze the nucleolytic intramolecular self-scission reactions (Figure 3) (reviewed in (Cochrane and Strobel, 2008a)). Their reaction mechanism are very similar, and they typically involve base catalyzed activation of the site-specific 2′-hydroxyl that acts as a nucleophile to attack the adjacent scissile 3′-phosphate. This reaction yields two RNA products: one containing the 2′,3′-cyclic phosphate and the other the 5′-hydroxyl functional group (Figure 3A). The only exception to this mechanism is the *glmS* ribozyme, which requires an external cofactor in the self-cleavage reaction (Cochrane et al., 2007).

The self-scission reactions catalyzed by these ribozymes are proposed to utilize the minor tautomeric form of catalytic guanosines, in which the N1 is not protonated, to act as a general base in the 2′-hydroxyl activation step of the reaction (Singh et al., 2015) (Figure 3A). Structural studies of hammerhead (Martick and Scott, 2006), hairpin (Rupert and Ferré-D'Amaré, 2001), VS (Suslov et al., 2015) and the glmS (Cochrane et al., 2007; Klein and Ferré-D'Amaré, 2006) ribozymes identified the N1 of catalytic guanosines in close proximity, within hydrogen bonding distance, to the 2′-hydroxyl nucleophile. These studies established that the N1 of G33 in *glmS* (Figure 3B) (Cochrane et al., 2007; Klein and Ferré-D'Amaré, 2006), G8 in hairpin (Figure 3C) (Fedor, 2000; Pinard et al., 2001; Rupert and Ferré-D'Amaré, 2001; Kuzmin et al., 2004; Bevilacqua and Yajima, 2006), G12 in hammerhead (Figure 3D) (McKay, 1996; Han and Burke, 2005; Martick and Scott, 2006; Thomas and Perrin, 2008), and G638 in VS (Figure 3E) (Lafontaine et al., 2001; Hiley et al., 2002; Sood and Collins, 2002; Suslov et al., 2015) play the role of a general base in the self-cleavage reactions catalyzed by these ribozymes.

However, the N1 of guanosine has a p*K*_a_ of ∼10 and is protonated at the physiological pH of ∼7.4 (Singh et al., 2015). The protonated N1 of guanosine is a poor base to abstract a proton from the 2′-hydroxyl, which has a p*K*_a_ of ∼13 (Velikyan et al., 2001). Therefore, tautomeric or ionic forms of the catalytic guanosines in which the N1 is not protonated are expected to form transiently during the catalysis. These minor tautomers are likely more nucleophilic at N1, and thus could extract the proton from the 2′-hydroxyl groups (Singh et al., 2015). Generation of N1 unprotonated guanosine would require perturbation of its p*K*_a_ towards neutrality. Significant perturbations in p*K*_*a*_, by as much as four units, have been reported in RNA systems, including ribozymes and riboswitches (Legault and Pardi, 1997; Wilcox and Bevilacqua, 2013). Perturbation of p*K*_a_ towards neutrality would facilitate deprotonation at the N1 site of catalytic guanosines because prototropic tautomerism involves the deprotonation and the protonation steps, and is optimal in functional groups whose p*K*_a_’s are close to neutral (Singh et al., 2015).

Despite significant structural and biochemical evidence in support for the presence minor tautomeric forms of catalytic guanosines in small self-cleaving ribozymes, their direct identification has proven challenging, owing to the lack of sensitive methods (Singh et al., 2015).

#### Tautomerism in DNA and its Role in Replication Fidelity

The genetic integrity of genomic DNA relies on adenine, guanine, cytosine and thymine existing predominantly in their keto and amino tautomeric forms during replication and transcription (Watson and Crick, 1953; Topal and Fresco, 1976; Wang et al., 2011; Rangadurai et al., 2020). Therefore, replication fidelity is expected to be influenced by the formation of minor tautomers (Watson and Crick, 1953; Topal and Fresco, 1976; Wang et al., 2011; Rangadurai et al., 2019; Rangadurai et al., 2020). In their work on the structure of DNA, Watson and Crick did appreciate that the formation of minor tautomeric forms would alter the base pairing properties of nucleic acid bases, potentially with mutagenic consequences (Watson and Crick, 1953). These minor tautomeric forms could arise from inter-helical transfer of protons in a DNA duplex (Figure 4) (Löwdin, 1963; Sevilla et al., 1995). Transient formation of minor tautomeric forms of DNA bases, and their stabilization in the active site of DNA polymerases during replication, could lead to incorporation of mismatched base pairs (Topal and Fresco, 1976; Watson and Crick, 1953; Wang et al., 2011). This phenomenon is plausible considering that the kinetics of minor tautomer formation and their lifetime in the active site is comparable with the kinetics of nucleotide incorporation by the polymerase. Therefore, the probability of base misincorporation during DNA replication may be correlated with the probability of rare tautomer formation (Figure 4A) (Topal and Fresco, 1976; Wang et al., 2011; Peng et al., 2015; Rangadurai et al., 2019).

Structural evidence for the rare tautomer hypothesis for spontaneous mutation came from the high-resolution crystal structure of a DNA polymerase that catalyzes replication in crystals (Figure 4B) (Wang et al., 2011). It was observed that a C•A mismatch mimics the shape of the cognate C•G base pair in the crystal (Wang et al., 2011). The movement of protons in the mismatched bases alter the hydrogen-bonding pattern such that the base pairs involving the minor tautomeric forms adopt an overall shape that is virtually indistinguishable from the canonical W-C base pair in DNA (Wang et al., 2011). This “shape mimicry” allows the mismatch to evade error detection mechanisms of human polymerases (Figure 4B). These observations provided structural support for the rare tautomer hypothesis of spontaneous mutagenesis.

NMR dispersion methods have allowed us to gain better understanding of tautomeric dynamics in DNA, and in RNA duplexes (Kimsey et al., 2018; Rangadurai et al., 2019; Rangadurai et al., 2020). Using W-C mismatches such as G-T or G-U, these studies established that G•T/U wobble mismatches exist in dynamic equilibrium between three distinct W-C mismatched base pairs within the DNA and RNA duplexes (Kimsey et al., 2018; Rangadurai et al., 2019; Rangadurai et al., 2020). The three distinct W-C mismatches include two tautomeric and one anionic species (Kimsey et al., 2018; Rangadurai et al., 2019; Rangadurai et al., 2020). The tautomeric forms were established using the chemical shifts of guanine N1 and thymidine/uridine N3. The chemical shifts, although consistent with G^enol^•T/U base pair (minor enol tautomer of G paired with the dominant keto tautomeric form of T or U) were partially skewed toward G•T^enol^/U^enol^ pairs (keto-amino tautomer of G paired with enol tautomer of T or U) (Kimsey et al., 2018). This skewness was interpreted as evidence for a rapid equilibrium between the major G^enol^•T/U and the minor G•T^enol^/U^enol^ tautomeric base pairs (Kimsey et al., 2018). The two rapidly exchanging tautomeric species (G^enol^•T/U⇌G•T^enol^/U^enol^) were also quantitated and found to be around 0.4% of the total population at neutral pH. Increased understanding of base pairing mismatches like G•T, G•U, and A•C that nevertheless adopt W-C like geometry through either tautomerization or ionization allowed us to appreciate that these mismatches appear to be more common (Kimsey et al., 2018; Rangadurai et al., 2019; Rangadurai et al., 2020). Stabilization of W-C mismatches through tautomerization, either in the template strand or in the incoming nucleotide, could allow the incorporation of mismatches during replication (Topal and Fresco, 1976; Wang et al., 2011; Rangadurai et al., 2020), and remains a compelling mechanistic explanation for spontaneous mutagenesis.

### Therapeutic Implications of Tautomeric Nucleosides

Nucleoside/nucleotide analogs enriched in minor tautomers are effective as antiviral agents against many viruses (Crotty et al., 2001; Baranovich et al., 2013; Li et al., 2014; Singh et al., 2015; Delang et al., 2018; Kabinger et al., 2021). The antiviral property of these analogs stem from their ability to induce mutations in viral genomes (Li et al., 2014; Delang et al., 2018; Gordon et al., 2021; Kabinger et al., 2021). Such nucleoside analogs are mutagenic, in part, because they form significant amounts of minor tautomeric forms, which enables them to engage in ambiguous base pairing, i.e., paring with more than one base (Li et al., 2014; Delang et al., 2018; Kabinger et al., 2021). The concept of specifically increasing the mutation rates of pathogens to drive their population collapse is particularly effective against retroviruses and riboviruses (Crotty et al., 2001; Anderson et al., 2004; Domingo et al., 2008; Manrubia et al., 2010; Domingo et al., 2012). These viruses have high mutation rates and their population lives close to the mutational threshold called the error catastrophe limit (ECL), a theoretical mutational rate above which producing viable viral progeny becomes impossible and leads to viral population collapse (Eigen, 2002). This phenomenon is called lethal mutagenesis. Additionally, unlike the human replicative polymerases that feature both high selectivity and high fidelity, the replication machinery of retroviruses is both promiscuous and error prone and thus, it can readily incorporate mutagenic nucleoside analogs (Anderson et al., 2004; Li et al., 2014; Kabinger et al., 2021). Therefore, these nucleoside analogs selectively mutagenize the viral genomes, while leaving the host (human) genome essentially untouched (Crotty et al., 2001; Anderson et al., 2004; Delang et al., 2018; Kabinger et al., 2021).

Nucleoside analogs that induce lethal mutagenesis are effective as antiviral drugs against many retroviruses and riboviruses, such as HIV, HCV, influenza virus and coronaviruses (Anderson et al., 2004; Baranovich et al., 2013; Li et al., 2014; Delang et al., 2018; Gordon et al., 2021 Kabinger et al., 2021). These viruses have high mutation rates and their replication machinery is error-prone (Watson and Crick, 1953; Crotty et al., 2001; Wang et al., 2011; Li et al., 2014; Rangadurai et al., 2020; Kabinger et al., 2021). The error prone nature of some viral polymerases allows incorporation of modified nucleosides (Crotty et al., 2001; Li et al., 2014; Peng et al., 2015; Kabinger et al., 2021). The 5-aza-5,6-dihydro-2′-deoxycytidine (KP1212) is an experimental drug candidate that targets HIV by increasing the mutation rate of the virus (Figure 5) (Harris et al., 2005; Mullins et al., 2011; Li et al., 2014). The antiviral efficacy of KP1212 stems from its ability to exist in multiple tautomeric states (Li et al., 2014; Peng et al., 2015). These tautomers can engage in ambiguous base pairings to induce mutagenesis in HIV (Li et al., 2014; Peng et al., 2015). Other antiviral drugs such as ribavirin for HCV (Crotty et al., 2001; Li et al., 2014), and favipiravir for influenza (Baranovich et al., 2013; Delang et al., 2018) are also efficacious due, in part, to their ability to exist in multiple tautomeric or rotameric forms and induce mutagenesis in viral genomes. Lethal mutagenesis has also been implicated as the main mechanism of action for the recently developed small-molecule therapeutics, such as molnupiravir from Merck, for severe acute respiratory syndrome coronavirus (SARS-CoV-2, the virus that causes Covid-19) (Kabinger et al., 2021; Zhou, 2021).

The mechanism by which KP1212 induces lethal mutagenesis in HIV has been studied extensively (Li et al., 2014; Peng et al., 2015). KP1212 is mutagenic, inducing G to A and A to G transition mutations (Figure 5) (Harris et al., 2005; Li et al., 2014). The mutagenicity of KP1212 is due, in part, to its ability to exist in multiple tautomeric or rotameric forms (Peng et al., 2013; Li et al., 2014; Peng et al., 2015). While KP1212 is considered a dC (deoxycytidine) analog, because it features the functional groups and the W-C face of dC, it has a near neutral pKa of ∼7, compared to p*K*_a_ of ∼4 for dC. As a consequence, KP1212 in solution exists in multiple tautomeric states (Li et al., 2014; Peng et al., 2015). These include keto-amino, keto-imino and its rotameric form, enol-amino and enol-imino and its rotameric form (Li et al., 2014). The enol-amino and enol-imino are the dominant tautomeric forms of KP1212, in contrast to the dominant keto-amino form observed for dC (Li et al., 2014; Peng et al., 2015). The alternative tautomeric forms of KP1212 are expected to have perturbed base pairing properties (Li et al., 2014), and thus contribute to the mutagenic properties of the base. KP1212 was shown to induce G to A and A to G mutations in the HIV genomes in cellular models, in preclinical rodents studies and in HIV patients who participated in the clinical trials for KP1212 (Harris et al., 2005; Mullins et al., 2011; Li et al., 2014). However, these studies also revealed that KP1212 is not mutagenic to human cells. Lack of mutagenicity in humans is likely due to the higher fidelity and selectivity of human DNA replicative polymerases (Mullins et al., 2011). Unlike dC, KP1212 has a saturated carbon center at the 6-position, which causes the base ring to be puckered (Li et al., 2014). This geometric distortion is significant enough to be selected against by the human polymerases. KP1212, therefore, exemplifies the features of an ideal lethal mutagen because it specifically induces mutations in HIV, driving viral population collapse, while sparing the human genome.

Molnupiravir is a broad-spectrum nucleoside analog drug that is efficacious against many viruses including the proofreading-intact SARS-CoV-2 coronavirus with a high genetic barrier to resistance (Gordon et al., 2021; Agostini et al., 2019). Its efficacy stems from its ability to induce lethal mutagenesis in SARS-CoV-2, during the early stages of Covid-19 (Figure 5) (Agostini et al., 2019; Kabinger et al., 2021; Gordon et al., 2021; Menéndez-Arias, 2021). Molnupiravir is an isopropyl ester prodrug of the ribonucleoside analog β-D-N^4^-hydroxycytidine (NHC) (Kabinger et al., 2021). It is currently in the phase-III clinical trial for Covid-19 (Kabinger et al., 2021). Biochemical studies using SARS-CoV-2 RNA-dependent-RNA polymerase (SARS-CoV-2 RdRp) have shown that the triphosphate form of NHC can be incorporated into RNA, albeit at a lower frequency compared to canonical nucleosides (Gordon et al., 2021; Menéndez-Arias, 2021). Once incorporated into the growing strand, it can be extended at its 3′-end. When present in the template strand it can form base pairs with G (NHC:G) or A (NHC:A) to induce G to A or A to G mutations (Figure 5) (Gordon et al., 2021). The NHC:A base pairs is more efficiently extended compared to NHC:G (Kabinger et al., 2021). Cellular studies have shown that NHC is 100-fold more active than ribavirin and favipiravir against SARS-CoV-2 (Zhou, 2021). The higher activity molnupiravir also induces a higher mutation frequency in the viral RNA (Menéndez-Arias, 2021; Zhou, 2021). Structural analysis of RdRp–RNA complexes containing mutagenesis products revealed that the NHC moiety can form stable base pairs with either G or A in the active site of RdRp (Kabinger et al., 2021). The formation of stable base mispairs with G or A, in W-C conformations, allow molnupiravir to escape the proofreading mechanism of the SARS-CoV-2 RdRp polymerase (Agostini et al., 2019; Gordon et al., 2021; Kabinger et al., 2021; Menéndez-Arias, 2021). The accumulation of mutations with each round of viral multiplication would lead to weakening of the virus (Kabinger et al., 2021; Menéndez-Arias, 2021). Similar to molnupiravir against SARS-CoV-2, ribavirin and favipiravir target HCV, and influenza, respectively, in part by the lethal mutagenesis mechanism (Crotty et al., 2001; Baranovich et al., 2013). Taken together, all the above studies underscore the importance of better understanding the mechanisms by which nucleoside analogs are incorporated and lead to mispaired bases in viral genomes.

### Methods for Studying Tautomerism in Nucleic Acids and in Nucleoside Analogs

**FIGURE 6:**
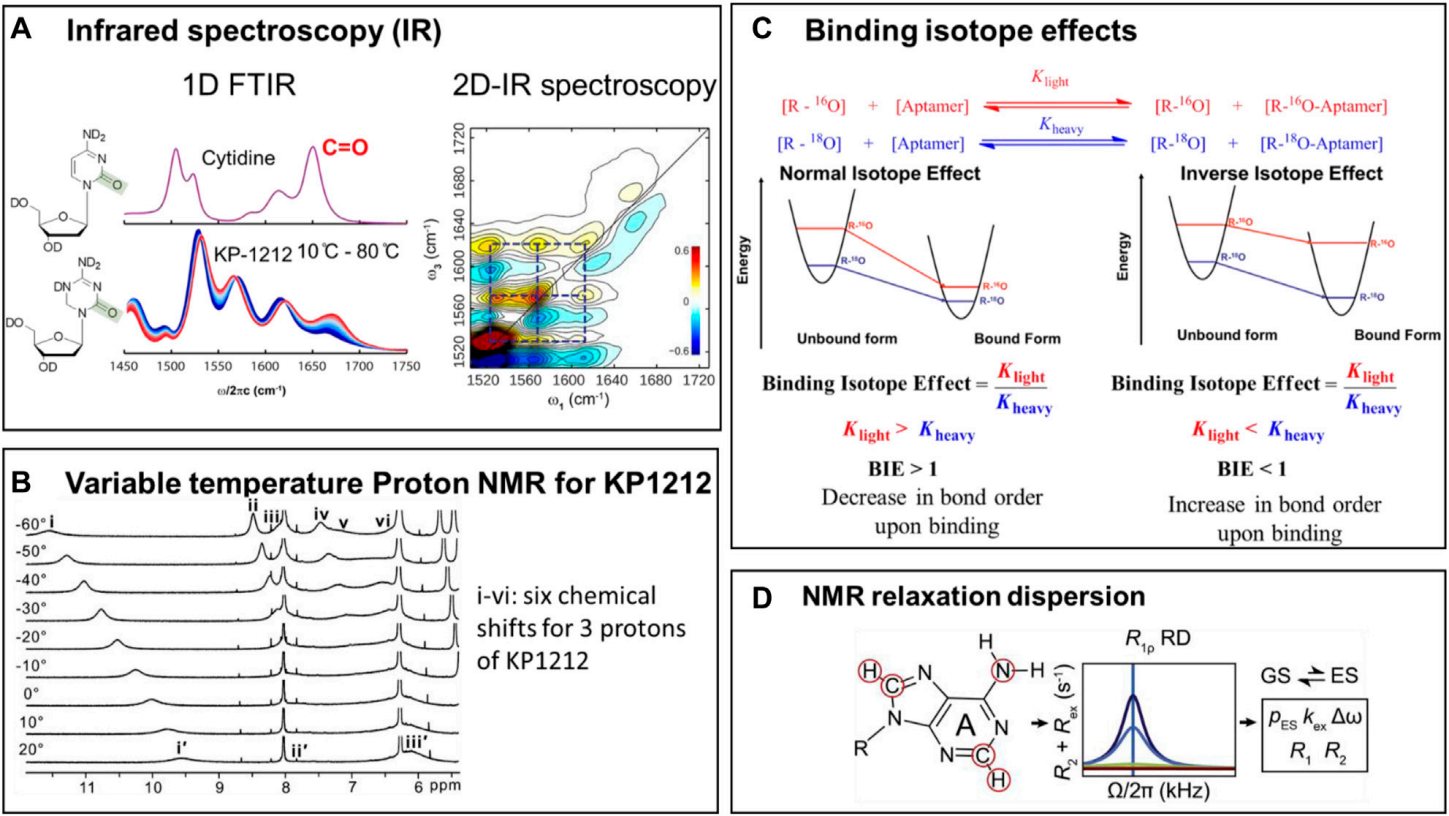
Methods for studying tautomerism in nucleoside analogs and nucleic acids. **(A)** Left side: The Fourier-Transform Infrared (FTIR) spectra for 2′-deoxycytidine (dC) at room temperature, and the temperature dependence of vibrational frequencies in the. FTIR spectra for KP1212. Right side: The 2-Dimensional Infrared (2D-IR) spectroscopic data for KP1212, in aqueous solution at room temperature (Li et al., 2014). **(B)** Variable temperature 1H-NMR data for KP1212 (Li et al., 2014). Figures 6A,B are adapted from (Li et al., 2014). **(C)** Binding isotope effects combined with Density Functional theory (DFT) calculations used in the characterization of the tautomeric form of OxyTPP bound to the TPP riboswitch (Singh et al., 2015). Reprinted (adapted) with permission from (Singh et al., 2014) *Copyright © 2014, American Chemical Society.* **(D)** NMR relaxation dispersion methods used to identify and quantitate rare tautomers in a G•T mismatch in DNA/RNA duplexes in W-C conformations (Rangadurai et al., 2019). Figure courtesy of Hashim Al-Hashimi Duke University.

Minor tautomers of canonical nucleic acid bases and their analogs are rare, transient, and interconvert on a fast time scale (Topal and Fresco, 1976; Rangadurai et al., 2019; Peng et al., 2015). Recent developments of spectroscopic methods have allowed their direct identification in nucleic acids and in nucleoside analogs (Figure 6) (Wang et al., 2011; Peng and Tokmakoff, 2012; Peng et al., 2013; Singh et al., 2014; Li et al., 2014; Peng et al., 2015; Szymanski et al., 2017; Rangadurai et al., 2019; Rangadurai et al., 2020; Kabinger et al., 2021). Prototropic tautomers differ from one another based on the position of protons, and the position of double bonds. Therefore, methods that are capable of detecting and distinguishing protons in different chemical environment (for example, NMR) are useful for the direct identification of tautomeric forms (Singh et al., 2014; Li et al., 2014; Peng et al., 2013) in both nucleic acids and nucleoside analogs (Figures 6B,C) (Li et al., 2014; Peng et al., 2015; Rangadurai et al., 2019; Rangadurai et al., 2020). Similarly, methods that provide information on the bond order and strength of a specific chemical bond (such as IR and Raman spectroscopy) are useful for distinguishing between keto-enol or amino-imino tautomeric forms, because the functional groups in each tautomeric pair have very different and characteristic vibrational properties. In addition, biochemical binding isotope effects and their interpretation using computational DFT have been used for characterizing tautomeric forms of a ligand in the binding pocket of an RNA aptamer (Figure 6C) (Singh et al., 2014).

NMR measures the chemical shift of individual protons, a property that depends on the chemical environment of the proton. Since the protons in tautomeric pairs are attached to different heteroatoms, their NMR chemical shifts are very different and thus distinguishable. Often, because the tautomeric equilibria are faster than the NMR acquisition time scale, broader peaks are observed, that span areas of multiple tautomeric forms. However, by substantially lowering the temperature of the sample (a method called variable temperature NMR), the tautomeric equilibria can be slowed down sufficiently to resolve the individual tautomeric forms.

Infrared spectroscopy (IR) measures the vibrational properties of the chemical bonds. Depending on their chemical makeup and bond order, different bonds absorb infrared radiation at different wavelengths. IR spectra provide direct information on the types of bonds present in a molecule, and therefore can easily distinguish between keto-enol or amino-imino tautomeric forms. Two-dimensional IR (2D-IR), an advanced version of IR, uses the same principle but provides, in addition, information on bond connectivity. Bonds in the same molecule vibrate together, and thus give a characteristic cross peak in the spectrum, which allows the distinction between the individual tautomeric forms present at equilibrium.

In addition to the direct detection of rare tautomers in nucleic acids, which is very challenging, indirect approaches, based on binding isotope effects (BIEs), exist that allow inference of minor tautomer formation. Theoretically, BIEs report on changes in vibrational frequencies of a ligand upon binding to its target. Experimentally, they are estimated using the binding affinities of ligands carrying either light or heavy isotopes of atoms directly involved in the binding interaction. The experimental BIEs are then compared to BIEs calculated, using the Density Functional Theory (DFT), from frequencies of optimized tautomeric forms. The exact characterization is completed by identifying a tautomeric structure whose calculated BIEs closely match the experimental BIEs. A good example of this approach is the identification of tautomeric form of OxyTPP recognized by the TPP riboswitch (Figure 6C), where spectroscopic observations indicated that each tautomer has a unique vibrational frequency pattern. This approach was used to study OxyTPP bound to the TPP riboswitch (Figure 6C), using ^18^O and ^32^P/^33^P labeled OxyTPP (Singh et al., 2014; Singh et al., 2015). Although the calculated BIEs are different for the keto and enol forms of OxyTPP, the magnitude of the difference between the BIEs corresponding to various tautomers of OxyTPP was too small to yield a definite conclusion. More precise measurements of BIEs are nevertheless expected to provide clarity on the tautomeric form of OxyTPP bound to the TPP riboswitch (Sood and Collins, 2002; Singh et al., 2014). These studies also underscore the potential of indirect measurements like BIEs and kinetic isotope effects (KIEs) to increase our understanding of tautomerism in nucleic acids and base analogs.

The variable temperature NMR, FT-IR (Fourier Transform Infrared) and 2D-IR (2-dimensional Infrared) methods were used to identify the tautomers of KP1212 and oxythiamine (Singh et al., 2014; Li et al., 2014; Peng et al., 2015). Tautomeric dynamics of these nucleoside analogs are fast, typically on the nanosecond timescale (Peng et al., 2013; Peng et al., 2015). Therefore, attempts to distinguish between the multiple tautomers of KP1212 at room temperature using NMR were not successful. Lowering the temperature to −20°C allowed identification of individual tautomers of KP1212 and oxythiamine by 1-dimentional proton NMR (^1^H-NMR) in the dimethylformamide (DMF) solvent (Figure 6B) (Singh et al., 2014; Li et al., 2014; Peng et al., 2015). While the characterization and distribution of tautomers of KP1212 and oxythiamine by ^1^H-NMR utilized non-physiological conditions with low temperature and an aprotic solvent (DMF) (Li et al., 2014), these experiments clearly outlined the chemical plausibility of the tautomeric forms of these compounds. For detecting multiple tautomers under physiologically relevant aqueous conditions at room temperature, IR-based methods have proven to be more effective (Singh et al., 2014; Li et al., 2014; Peng et al., 2015). The temperature dependence of change in amplitude of vibrational frequencies in the 1D-FTIR spectrum confirmed the presence of multiple tautomers for KP1212 and oxythiamine (Figure 6C) (Li et al., 2014). To directly identify minor tautomers based on altered vibrational frequency of minor tautomers, 2D-IR data was combined with DFT calculations (Figure 6A) (Peng et al., 2015). The 2D IR spectrum has a greater spatial and temporal resolution than FTIR, and thus allows direct identification of multiple fast interconverting tautomers (Peng et al., 2013; Li et al., 2014; Peng et al., 2015). These studies established that KP1212 exists in seven different tautomeric or rotameric forms and oxythiamine exists in three different tautomeric forms (Li et al., 2014; Singh et al., 2014). Furthermore, the biochemical observations that KP1212 is 10% mutagenic when replicated *in vitro* and in living cells, and induces G to A mutations, can be rationalized by the tautomeric distribution identified from these spectroscopic studies (Li et al., 2014; Peng et al., 2015).

By contrast with the nucleoside analogs described above, the minor tautomers of the canonical DNA bases are significantly less abundant, shorter lived and more challenging to detect (Peng et al., 2013; Rangadurai et al., 2019; Peng et al., 2015). NMR relaxation dispersion (RD) methods allow identification of low-abundance short-lived conformational states in biomolecules (Figure 6D) (Mulder et al., 2001; Rangadurai et al., 2019; Rangadurai et al., 2020). NMR relaxation dispersion (RD) has been used to characterize rare tautomers in Hoogsteen base pairs versus Watson-Crick base pairs in different types of DNA/RNA, such as A or B type of DNA/RNA (Nikolova et al., 2011; Alvey et al., 2014; Zhou et al., 2016; Rangadurai et al., 2018). These results offer understandings into differences between A-RNA and B-DNA duplexes and provide possible explanations for how they respond to damage and modifications.

Using NMR RD, it has been shown that the G•T/U mismatches exist in dynamic equilibrium between tautomeric and anionic W-C conformations within the DNA and RNA duplexes. These studies also provided insights into the kinetics of rare tautomer formation in nucleic acids, and the probability of base misincorporation due to the formation of minor tautomers (Rangadurai et al., 2019; Rangadurai et al., 2020). These methods continue to enhance our understanding of the mechanisms by which the formation of minor tautomers contribute to the generation of spontaneous mutations.

Structural methods based on X-ray crystallography and Cryo-EM (Cryogenic electron microscopy) have proven effective in studying mismatched base pairs that potentially involve minor tautomers. While protons are very difficult to visualize directly by X-ray crystallography and Cryo-EM, the overall geometry of base pairs and the inter-heteroatom distances can be accurately measured, and thus allow to infer the presence of protons and hydrogen bonding interactions. Specifically, crystallographic studies have shown that mismatches in high fidelity DNA polymerases can adopt structures that closely mimic the W-C base pairing geometry (Wang et al., 2011; Kabinger et al., 2021). The existence of mismatches in conformations that are identical to W-C shapes may allow them to evade the polymerase proof reading mechanisms and lead to mutagenesis (Figure 4B, Figure 5A) (Bebenek et al., 2011; Wang et al., 2011; Fedeles et al., 2015; Kabinger et al., 2021). In sum, these studies have provided convincing structural evidence in support of rare tautomer hypothesis for the generation of spontaneous mutations that arise during nucleic acid replication.

## Future Directions

The last decade has seen significant progress in our understanding of tautomerism in DNA and RNA. Despite the early realization that tautomerism could influence nucleic acid biology, directly identifying rare tautomers of nucleic acid bases proved very challenging. The minor tautomers of DNA bases are present at equilibrium in minute amounts (< 0.1%) and their dynamics of interconversion is generally very fast. Until recently, little progress was made in directly visualizing minor tautomers of DNA bases. The NMR RD allowed direct detection of rare tautomers and characterized the dynamics of their formation in short DNA sequences. Structurally establishing that mismatches can be stabilized in W-C conformation by high fidelity DNA polymerases, provided crystallographic support for the rare tautomer hypothesis of spontaneous mutations. Advancement in methods based on BIEs, high-resolution crystallography, NMR and IR significantly enhanced our mechanistic understanding of mutagenesis from nucleoside analogs based antiviral drugs. While all these advancements are notable, they only reflect special cases of tautomerism in a limited set of experimental conditions. Much work remains to develop more broadly applicable methods that enable the direct visualization and identification of minor tautomers of canonical bases in complex biological systems, such as genomic DNA, and in the binding pocket of polymerases, under physiological conditions. Sensitivity improvements in all the methods summarized above are all expected to improve our understanding of tautomerism. In the case of BIEs, more precise measurements are expected to help identify minor tautomers in the context of RNA systems and may provide further insights into the role of tautomerism in RNA biology, including catalytic processes and ribozymes. More work is also needed to bridge the knowledge gap between the in-solution tautomeric equilibria and dynamics of nucleobases and analogs, and their biological consequences. In the case of antiviral nucleoside analogs, such as KP1212 and molnupiravir, there is great interest to understand how tautomeric equilibria translate into mutagenic potential inside the viral polymerases. These nucleoside analogs have already proven very effective as antiviral drugs. However, our understanding of the physico-chemical properties that enable these compounds to adopt multiple tautomeric forms is still limited. Further insight into tautomeric equilibria and dynamics should allow us to develop more potent and safer antiviral therapeutics.
